# Fox Serum Proteomics Analysis Suggests Host-Specific Responses to *Angiostrongylus* *vasorum* Infection in Canids

**DOI:** 10.3390/pathogens10111513

**Published:** 2021-11-19

**Authors:** Nina Gillis-Germitsch, Tobias Kockmann, Christian M. O. Kapel, Stig M. Thamsborg, Pia Webster, Lucienne Tritten, Manuela Schnyder

**Affiliations:** 1 Institute of Parasitology, Vetsuisse Faculty, University of Zurich, Winterthurerstrasse 266a, 8057 Zurich, Switzerland; nina.gillis-germitsch2@uzh.ch; 2 Graduate School for Cellular and Biomedical Sciences, University of Bern, Mittelstrasse 43, 3012 Bern, Switzerland; 3 Functional Genomics Center Zurich, Swiss Federal Institute of Technology Zurich (ETH Zurich), University of Zurich, Winterthurerstrasse 190, 8057 Zurich, Switzerland; tobias.kockmann@fgcz.ethz.ch; 4 Section for Organismal Biology, Department of Plant and Environmental Sciences, University of Copenhagen, Thorvaldsensvej 40, 1871 Frederiksberg C, Denmark; chk@plen.ku.dk; 5 Veterinary Parasitology Research Group, Department of Veterinary and Animal Sciences, University of Copenhagen, Dyrlægevej 100, 1870 Frederiksberg C, Denmark; smt@sund.ku.dk (S.M.T.); PIW@ssi.dk (P.W.); 6 Prediagnostics and Service, Infectious Disease Preparedness, Statens Serum Institut, Artillerivej 5, 2300 Copenhagen S, Denmark

**Keywords:** *Angiostrongylus vasorum*, proteomics, fox, *Vulpes vulpes*, immune response, coagulation

## Abstract

Dogs infected with the cardiopulmonary nematode *Angiostrongylus vasorum* may suffer from respiratory distress and/or bleeding disorders. Descriptions of clinical signs in foxes are rare, despite high prevalence. To evaluate the impact of infection on coagulation and immune response, serum proteins from eight experimentally infected foxes before and after inoculation (day 0, 35, 84, 154) were subjected to differential proteomic analyses based on quantitative data and compared to available data from dogs. The number of proteins with differential abundance compared to the uninfected baseline increased with chronicity of infection. Bone marrow proteoglycan, chitinase 3-like protein 1 and pulmonary surfactant-associated protein B were among the most prominently increased proteins. The abundance of several proteins involved in coagulation was decreased. Enriched pathways obtained from both increased and decreased proteins included, among others, “platelet degranulation” and “haemostasis”, and indicated both activation and suppression of coagulation. Qualitative comparison to dog data suggests some parallel serum proteomic alterations. The comparison, however, also indicates that foxes have a more adequate immunopathological response to *A. vasorum* infection compared to dogs, facilitating persistent infections in foxes. Our findings imply that foxes may be more tolerant to *A. vasorum* infection, as compared to dogs, reflecting a longer evolutionary host–parasite adaptation in foxes, which constitute a key wildlife reservoir.

## 1. Introduction

*Angiostrongylus vasorum* is a cardiopulmonary nematode of canids. Adults reside in the right side of the heart of the definitive hosts, where females release numerous eggs. First-stage larvae (L1) hatch and migrate through the lung where they cause tissue damage and pneumonia. First-stage larvae are eventually coughed up, swallowed, and excreted with the faeces. Slugs and snails act as intermediate hosts, in which L1 develop to infectious third-stage larvae (L3). Canids are infected by the ingestion of L3 contained in gastropod intermediate hosts, which then migrate to the heart where they further develop [1]. *Angiostrongylus vasorum* is endemic in Europe and parts of North and South America [2,3,4]. In Europe, incidence rates in dogs and prevalence in foxes increased in the last few decades in countries where *A. vasorum* has been present for many years [5,6,7] and, simultaneously, has been increasingly detected in areas where it has not been reported before [8,9,10]. In the domestic cycle, the main definitive hosts are dogs (*Canis lupus familiaris*). In Europe, foxes (*Vulpes vulpes*) serve as the most important reservoir host, but depending on the geographical region, other wild canids such as coyotes (*Canis latrans*) [11], crab-eating foxes (*Cerdocyon thous*) [12], hoary foxes (*Dusicyon* *vetulus*) [13] or wolves (*Canis lupus*) [14] and other animals such as red pandas (*Ailurus fulgens fulgens*) [15], badgers (*Meles meles*) [16], and meerkats (*Suricata suricatta*) [17] may serve as definitive hosts.

The clinical picture of canine angiostrongylosis is broad and highly variable [2,18]. Infected dogs may suffer respiratory distress with, e.g., coughing and dyspnoea [19], bleeding disorders, which manifest in internal or external bleeding [20,21,22], or may show neurological signs due to haemorrhages in the central nervous system [23]. In foxes, clinical signs are rarely described [24,25], and experimental infections with 50 or 200 L3 did not lead to clinical illness [26,27]. However, diagnostic imaging and post mortem investigations of infected dogs and foxes indicated severe lung pathologies in both species [5,19,28,29,30,31]. Today, the differences in clinical manifestations and in the host–parasite interactions between *A. vasorum* infected dogs and foxes remain largely unstudied and poorly understood. A limited number of studies with a focus on the immune response or coagulation status have been conducted in dogs [19,22,32,33]. To improve our understanding of the host–parasite interplay between foxes and *A. vasorum*, and to assess whether serum protein alterations observed in *A. vasorum*-infected foxes are host-specific, we performed fox serum proteome analysis using experimental samples and compared the data to those reported recently in dogs [32].

## 2. Results

Serum samples from eight *A. vasorum* experimentally infected foxes were drawn at four different time points (right before inoculation (d0), and during prepatency (d35), patency (d84) and the chronic stage of infection (d154, 5 foxes only)) and analysed by nano liquid chromatography coupled to mass spectrometry (LC-MS) to monitor the compositional changes of the blood proteome in response to parasite infection. In total, 751 proteins were identified and quantified across all analysed samples (Supplementary Figure S1), 4 of which were identified as *A. vasorum* proteins.

### 2.1. Differentially Abundant Proteins

**Figure 1:**
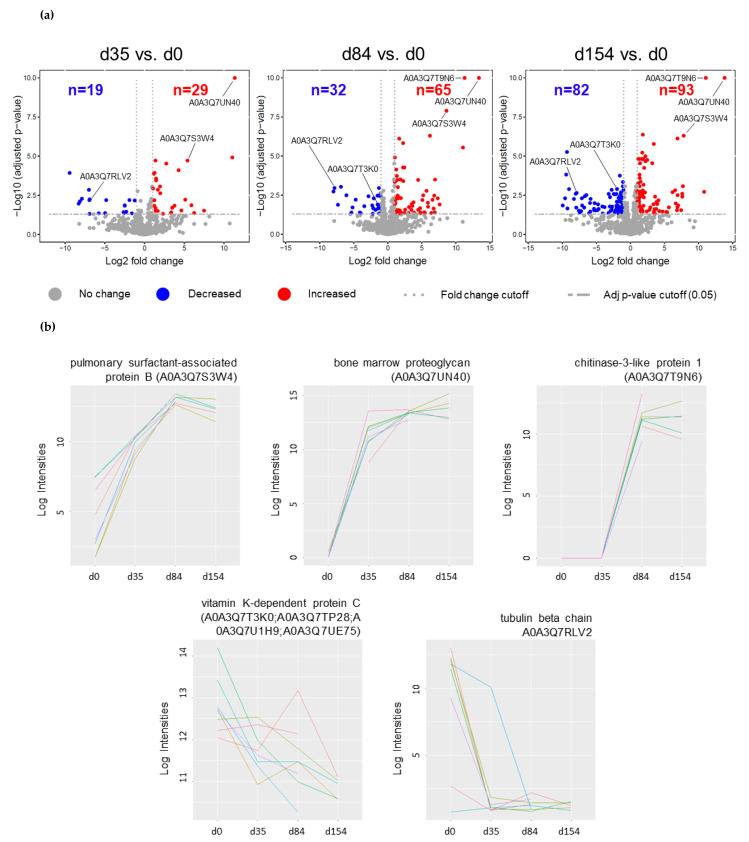
(**a**) Volcano plots of significantly increased and decreased fox serum proteins (fold change cut-off ≥ 1, or ≤ −1) of up to 8 *Angiostrongylus vasorum* experimentally infected animals. Three time points of infection (d35, d84, d154) were compared to before infection (d0). (**b**) Individual profile plots for selected serum proteins of infected foxes: pulmonary surfactant-associated protein B (A0A3Q7S3W4), bone marrow proteoglycan (A0A3Q7UN40), chitinase-3-like protein 1 (A0A3Q7T9N6), vitamin K-dependent protein C (A0A3Q7T3K0; A0A3Q7TP28; A0A3Q7U1H9; A0A3Q7UE75), and tubulin beta chain (A0A3Q7RLV2). On the *x* axis are the time points d0, d35, d84, and d154, and log2 protein intensities are listed on the *y* axis.

Overall, 276 proteins showed significant abundance increase or decrease (log2 fold change (FC) ≥ 1/≤ −1, adjusted *p*-value ≤ 0.05) with respect to d0. Among them were two *A. vasorum* proteins, which were excluded from further analysis. A detailed list of all significantly increased or decreased proteins at all time point comparisons, including log2 FC and adjusted *p*-values, can be found in Supplementary Table S1. Compared to before infection (d0), levels of 29 proteins were increased at d35, while 19 were decreased. At d84, compared to before infection (d0), the abundance of 65 proteins was increased and the abundance of 32 proteins decreased. Of these, 40 and 20 proteins were not significantly altered earlier, at d35, and therefore, additionally increased and decreased, respectively. One hundred fifty-four days after inoculation, 93 proteins were increased and 82 were decreased compared to d0 (Figure 1a). Among increased and decreased proteins, 48 and 57 proteins had not been significantly altered at previous time points, respectively. Comparing d84 to d35, the abundance of 17 proteins had either increased (12) or decreased (5), and comparing d154 to d35, levels of 140 proteins (88 and 52, respectively) were changed. Comparing d154 to d85, the abundance of 42 proteins had increased (16) or decreased (26).

The most strongly increased protein across all time point comparisons was bone marrow proteoglycan (A0A3Q7UN40; FC: 11.4–13.8). Other significantly and highly increased proteins as of d85 (compared to before infection) were chitinase-3-like protein 1 (A0A3Q7T9N6), pulmonary surfactant-associated protein B (A0A3Q7S3W4), and interleukin enhancer-binding factor 2 (A0A3Q7SCP6; A0A3Q7TCE1). Further significantly increased proteins involved in immune response, inflammation or coagulation included, among others, fibrinogen alpha and beta chains (A0A3Q7T5Z6/A0A3Q7TRK4), fibulin 2 and 5 (A0A3Q7RP65; A0A3Q7SXX8/A0A3Q7R7A1), thrombospondin 1 and 3 (A0A3Q7SDJ4/A0A3Q7SAF2), and eosinophil peroxidase (A0A3Q7S7C1). Among the most decreased proteins were tubulin beta chain proteins (A0A3Q7UCJ4/A0A3Q7RLV2), LIM and senescent cell antigen-like-containing domain protein (A0A3Q7TMY6; A0A3Q7UH09), beta-parvin (A0A3Q7U5D8), and integrin-linked protein kinase (A0A3Q7RAD1). Further significantly decreased proteins involved in immune response, inflammation or coagulation included coagulation factor XIII A chain (A0A3Q7RD39), vitamin K-dependent protein C (A0A3Q7T3K0; A0A3Q7TP28; A0A3Q7U1H9; A0A3Q7UE75), macrophage migration inhibitory factor (A0A3Q7QZI1), coagulation factor X (A0A3Q7SQ30), and coagulation factor IX (A0A3Q7UBG7). Changes were mostly consistent across all eight foxes. Figure 1b displays five examples.

### 2.2. Gene Ontology and Pathway Enrichment

Table 1 and Table 2 display the top 10 gene ontology biological processes and significantly enriched pathways for each time point comparison to before infection (d0) resulting from increased or decreased proteins, respectively. All significant biological processes and enriched pathways from either increased or decreased proteins are listed in Supplementary Table S2.

Among the top 10 biological processes resulting from significantly increased proteins (adjusted *p*-value ≤ 0.05) in infected foxes compared to uninfected baseline, considering all time point comparisons combined, were “neutrophil mediated immunity” (GO:0002446), “neutrophil activation involved in immune response” (GO:0002283), “neutrophil degranulation” (GO:0043312), and “extracellular matrix organization” (GO:0030198). Further biological processes resulting from GO enrichment from both increased and decreased protein sets were, e.g., “platelet degranulation” (GO:0002576), “plasminogen activation” (GO:0031639), and “positive regulation of coagulation” (GO:0050820).

Among enriched pathways resulting from gene set enrichment analysis based on increased proteins (all time points combined) were “extracellular matrix organization”, “platelet degranulation”, “formation of fibrin clot (clotting cascade)”, “platelet activation, signalling and aggregation”, and “haemostasis”. “Platelet degranulation”, “response to elevated platelet cytosolic Ca^2+^”, “intrinsic pathway of fibrin clot formation”, and “haemostasis” were among enriched pathways obtained from decreased proteins.

### 2.3. Qualitative Comparison of Serum Alterations of A. vasorum Experimentally Infected Foxes and Dogs

**Figure 2:**
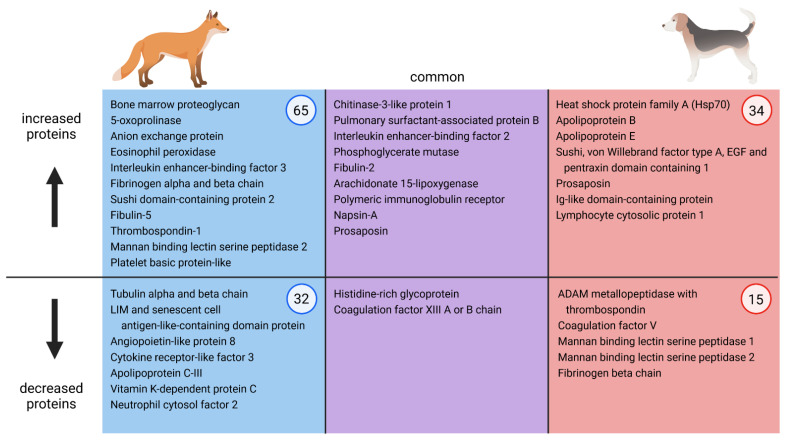
Comparison of abundant common and diverging increased and decreased serum proteins involved in immune response, inflammation, or coagulation of *Angiostrongylus vasorum* experimentally infected foxes and dogs at d84 vs. d0 and d75 vs. d-7, respectively (the overall number of in- or decreased proteins at this time point comparison are encircled). Dog data was obtained from Tritten et al. [32]. This figure was created using BioRender.com.

Significantly increased or decreased fox serum proteins were qualitatively compared to those found in an experimental dog infection model, where comparable time points were examined, i.e., d-7, d34, d75, d104, and d230 [32]. Applying the same cut-off at adjusted *p*-value ≤ 0.05 and log2 FC ≥  1, or ≤ −1, foxes had a larger number of increased or decreased proteins at each time point compared to dogs. Bone marrow proteoglycan, the most prominently increased protein in fox samples at each time point after onset of infection, was not increased in dogs. At day 35, both dogs and foxes showed increased abundance of pulmonary surfactant-associated protein B, polymeric immunoglobulin receptor, and CD5 antigen-like protein. At d75, the most strongly increased protein in dogs was chitinase 3-like protein 1, which was the second most prominently increased protein in foxes at d84. At the same time point, pulmonary surfactant-associated protein B was the second most strongly increased protein in dogs and the fourth most strongly increased in foxes. Other proteins with increasing abundance over the course of infection common in both species at d84 vs. d0 (foxes) or d75 vs. d-7 (dogs) were, among others, fibulin 2, interleukin enhancer-binding factor 2, prosaposin, and phosphoglycerate mutase (Figure 2). Chitinase 3-like protein 1 remained the most prominently increased protein at later time points in dogs and remained the second most increased protein at d154 in foxes. The same applies for pulmonary surfactant-associated protein B, which stayed the second most strongly increased in dogs and the fourth most prominently increased in foxes at the chronic stage of infection. Fibulin 2 abundance remained increased until the end of the experiment in both species, and phosphoglycerate mutase abundance only in foxes. The von Willebrand factor type A level was increased in both dogs and foxes at d230 and d154, respectively. In dogs, this protein had already been increased at d75, but not in foxes (d84). In dogs, levels of several Ig-like domain-containing proteins were increased over the course of infection, and coagulation factor VIII at d230, while these were not increased in foxes at any time points.

Among proteins at d35 and d34 compared to pre-infection, dogs and foxes had no decreased proteins in common. When comparing d84 vs. d0 in foxes with d75 vs. d-7 in dogs, the only decreased proteins present in both species were coagulation factor XIII (A chain in the fox and B chain in the dog) and histidine-rich glycoprotein (Figure 2). Comparing later time points to uninfected baseline (d104 vs. d-7 and d230 vs. d-7 for dogs and d154 vs. d0 for foxes), the only common decreased proteins were coagulation factor X, attractin, histidine rich glycoprotein, and adiponectin. Dogs had several decreased mannan binding lectin serine peptidases as of day 34 after infection; in foxes, mannan binding lectin serine peptidase 2 abundance was increased at d84 vs. d0 (Figure 2). Further dog serum proteins that were decreased at several time points but increased in foxes as of day 35 were thrombospondins and fibrinogen chains.

## 3. Discussion

Little is known about the effect of *A. vasorum* infection on foxes despite their important role as wildlife reservoir hosts [5]. The goal of this study was to identify serum alterations over time in foxes experimentally infected with *A. vasorum* to generate a deeper understanding of the host–parasite interaction between foxes and *A. vasorum.* A similar analysis over the course of *A. vasorum* infection has already proven useful to better understand the pathogenesis in dogs [32]. This allowed emphasizing differences in serum proteomes between *A. vasorum*-infected foxes and dogs. So far, no blood analyses have been carried out using naturally infected wild foxes. Two studies were conducted with *A. vasorum* experimentally infected foxes previously, focused on differences in larval excretion, worm burden, clinical and haematological alterations in juvenile and adult animals following single and repeated challenge infections [26,27]. Standard haematology, blood chemistry and coagulation analysis performed in one of these studies revealed eosinophilia, increased D-dimers and decreased platelets upon infection [26]. This prior work relied on fox samples of which only eosinophil counts were determined [27].

Our findings generated by LC-MS demonstrated that a high number of proteins show altered levels in infected foxes, with numbers increasing as infection progresses. The number of increased or decreased proteins in foxes (276) was twice as high as in infected dogs (139 proteins) [32]. Several proteins associated with inflammatory and immune response and proteins involved in coagulation were either increased or decreased in foxes, compared to the established pre-infection baseline. To further validate the obtained fox data, targeted protein quantification by Parallel Reaction Monitoring (PRM), as previously conducted for dog serum samples [32], could be conducted.

The protein that increased the most throughout experimental infection in foxes was bone marrow proteoglycan, also known as eosinophil major basic protein. This protein originates from eosinophils, key immune cells in the host defence against helminths [34]. Bone marrow proteoglycan acts as a cytotoxin and helminthotoxin; it causes activation and degranulation of neutrophils in a non-cytotoxic fashion [35,36], which is reflected in the enriched biological processes “neutrophil mediated immunity”, “neutrophil activation involved in immune response”, and “neutrophil degranulation”. Bone marrow proteoglycan further leads to activation and aggregation of platelets [37], contributing to the enriched pathway “platelet degranulation”. Bone marrow proteoglycan is usually released at the site where it is needed and can cause both a toxic and proinflammatory reaction [35]. Therefore, the 22- to 26-fold increase in this protein in foxes throughout the course of the infection indicates a strong innate immune response against *A. vasorum* proceeding from eosinophils and reflects findings of eosinophilia in experimentally infected foxes: eosinophilia has been reported in experimentally infected juvenile and adult foxes starting 3 and 4 weeks post infection and represented the only increased white blood cells upon infection [26]. In foxes from the current study, eosinophilia was shown to be present from d35 onwards, remaining until the end of the study (22 weeks after infection) [27]. In contrast, in experimentally infected dogs, eosinophilia was absent or mild until 90 days after infection [19], and was occasionally reported in naturally infected dogs [18,38]. Accordingly, bone marrow proteoglycan was not increased in serum used for quantitative proteomic analysis of experimentally infected dogs [32]. These findings, combined with clinical signs predominantly occurring in dogs, may indicate that this protein favours subclinical disease in foxes. Both dogs and foxes, however, manifest severe lung pathologies upon infection, suggesting that bone marrow proteoglycan does not protect from lung damage. The data may also suggest that foxes develop a more appropriate innate immune response to *A. vasorum* infection compared to dogs, leading them to better cope with infection, despite persisting infections [27]. Dogs, in turn, apparently fail to mount an appropriate and effective eosinophil response [19,32].

Further highly increased proteins at chronic stages of infection in both host species were chitinase-3-like protein 1 and pulmonary surfactant-associated protein B. Chitinase-3-like protein 1 is released by different cells, including neutrophils and macrophages. Inflammation induces an increase in chitinase-3-like protein 1 in the lung, where it is described to play a role in antibacterial responses and Th2 immune response, and increases mucin expression [39]. It has also been described to have antiparasitic properties through IL-17-mediated neutrophil recruitment as a response to nematode migration through lung tissue, leading to further lung damage [40].

Pulmonary surfactant-associated protein B is responsible for reducing the air surface tension in the lung alveoli, modulates the immune response and has some antimicrobial effects [41]. The increase in both of these proteins further reflects the immune and inflammatory response of the fox to *A. vasorum* infection. Indeed, L1, which hatch in pulmonary capillaries, migrate into the lung alveoli causing tissue damage, and lead to verminous pneumonia [19]. This process contributes to inflammation and likely induces an increase in both chitinase-3-like protein 1 and pulmonary surfactant-associated protein B. Both proteins were also detected at increased levels in *A. vasorum*-infected dogs and, therefore, were proteins with the strongest abundance increase upon infection in both species. This suggests that both dogs and foxes experience lung inflammation and damage to a comparable extent, which is also reflected in comparable lung pathologies in naturally and experimentally infected animals: consolidated lungs, nodule formation, discoloration, lung fibrosis and/or foci of chronic thrombosis, most pronounced at the margins of the lung lobes, are described for both dogs and foxes [5,19,28,29].

A few proteins that may influence coagulation or interact with endothelium, e.g., fibrinogen, thrombospondin 1, or fibulin 5, were increased in foxes. Their increase is mostly associated with inflammation [42,43]. Several coagulation factors and proteins involved in the coagulation cascade, such as vitamin K-dependent protein C, coagulation factor XIII A chain, coagulation factor IX, and coagulation factor X, however, were among decreased proteins in foxes, although none of them were decreased further than log2 FC < −2. Decreased abundance of coagulation proteins may represent a compensatory mechanism to increased abundance of proteins that may directly or indirectly activate coagulation. Interestingly, coagulation factor XIII A chain was decreased in foxes and, accordingly, its B chain in dogs [32]. Coagulation factor XIII stabilizes fibrin clots, and its deficiency can cause haemorrhages [44]. Mutations in the gene encoding A chains cause reduced fibrin clot thickness and result in clots more prone to degradation, eventually leading to increased bleeding tendencies [45]. This has, however, never been observed in foxes. Fibrinogen chains and thrombospondin 1 levels were increased in foxes but decreased in dogs. Both analyses in dogs and foxes were based on serum and not plasma samples, which generally should be depleted of fibrinogen and most coagulation factors or may only contain trace amounts. This may limit the interpretation of findings on some coagulation proteins. In addition, variations at different time points could be potentially due to differing clotting efficiency or clotting time before centrifugation or handling of the samples, which may have been slightly inconsistent. The same needs to be kept in mind when comparing altered coagulation components in dogs and foxes: differing blood collection systems may also have had an influence on clotting efficiency. Further, proteomes and genomes of both species are of different quality, and diverging annotations may have influenced the functional characterization of some proteins. Nonetheless, we observed that several proteins relevant for coagulation were either increased or decreased in foxes, leading to the enrichment of several coagulation pathways represented from both increased and decreased protein fractions, e.g., “platelet degranulation”, “platelet activation, signalling and aggregation”, “formation of fibrin clot (clotting cascade)”, “common pathway of fibrin clot formation”, and “haemostasis”. We hypothesize that *A. vasorum* infection may lead to both activation and suppression of coagulation in foxes, either through alterations induced by the parasite or through the host response. Indeed, blood dwelling parasites are known to interfere with the host coagulation in a balanced manner to neither induce nor suppress coagulation, which guarantees their long-term survival in the host [46].

Only one mannan binding lectin serine peptidase was increased in foxes, whereas several mannan binding lectin serine peptidases were decreased in dogs. Therefore, the complement cascade was among putative impaired pathways in dogs [32], which accordingly was not observed in foxes, indicating less involvement of the complement system in infected foxes. Another difference between the two canid species was the increase in Ig-like domain-containing proteins in dogs only, suggesting lower levels of circulating antibodies upon infection in foxes compared to dogs. This is in accordance with highly variable serum antibody responses among foxes and decreasing antibody levels within 7 weeks after infection (or repeated infections), despite high worm burdens in most individuals [26,27,47]. Declining levels of serum antibodies were observed in an earlier study in experimentally infected foxes [26]. In contrast, a strong and persisting antibody response was detected in dogs [48]. This may suggest that foxes develop tolerance to *A. vasorum*, but not without experiencing serum protein alterations. Particularly juvenile animals with high worm burdens showed more pronounced pathophysiological changes, especially regarding the coagulation parameter D-dimer, which is an indicator of active coagulation and fibrinolysis [26]. Seven out of eight foxes in the study presented here were juveniles. Naturally infected young foxes were previously found to have more severe lung pathologies as compared to adult animals [49]. The observed pathologies, however, were not reflected in a corresponding clinical picture, since no clinical signs were observed in experimentally infected foxes [26,27], and clinical signs have been very rarely reported in naturally infected wild foxes [24,25]. The apparent absence of clinical signs in foxes can most likely be explained by the intrinsic characteristics of wild animals: foxes are generally not held as pets or farm animals and, therefore, not monitored; in addition, they are known to have a much shorter life span [50,51], implying lower chances of establishment of chronic infections. But even though coagulopathies have never been observed in foxes, our findings suggest that the parasite may cause health impairment in foxes: an infection may shorten their life expectancy and/or increase their odds of succumbing to other diseases or accidents.

For the present study, farmed foxes bred for years in captivity and unexposed to *A. vasorum* were used. The used infective dose in foxes did not lead to clinical illness, despite proven constant larval excretion and corresponding worm burdens. Their strong eosinophil response and increased levels of bone marrow proteoglycan in sera may have contributed to the absence of clinical disease development. In addition, the variable individual antibody response without persistence of specific antibodies explains their susceptibility to reinfection and may indicate a fox-specific immunological response. Dogs in comparison mostly did not mount an eosinophil response and, accordingly, bone marrow proteoglycan levels were not increased in experimentally infected dogs. Experimentally and naturally infected dogs suffered from severe disease upon infection, despite persisting antibodies. Our findings, therefore, imply that *A. vasorum* is likely more adapted to foxes, and that foxes seem to cope better with the infection. This difference may be even more pronounced in wild foxes as compared to foxes farmed for generations. Therefore, under natural settings, the more adequate immune response of foxes could facilitate fox survival and, therefore, the spread of *A. vasorum* to larger areas, further supporting their importance as wildlife reservoir hosts.

## 4. Materials and Methods

### 4.1. Sera from Foxes Experimentally Inoculated with A. vasorum

Sera were taken from 1 adult and 7 juvenile female foxes experimentally infected with *A. vasorum* originating from a previously conducted experiment [27]. Briefly, foxes were inoculated with 100 *A. vasorum* L3, which were obtained from experimentally infected *Biomphalaria glabrata* snails. The isolate originated from an infected dog and had been passaged once in a fox. Well-being and potential clinical signs were monitored daily, and foxes were examined more thoroughly at blood sampling and before necropsy. Blood samples were obtained from the jugular veins (vacutainer-system SST tubes with clot activator) the day of inoculation and 35, 49, 63, 70, 84, 91, 98, 112, 119, and 140 days post inoculation (dpi), and on the day of necropsy (84 dpi, *n* = 3; 154 dpi, *n* = 5). Four to 8 h after sampling, sera were isolated by centrifugation (1380× *g*, 10 min) and kept at −20 °C until use. Differences between the euthanasia dates were related to the experimental design of the previously performed study. Detailed information on the experimental procedures, larval shedding, worm burden after necropsy, and haematology is available in Woolsey et al. [27]. Briefly, serum samples used for this study were from foxes with worm burden ranging from 18 to 61 (mean 55, with one out layer above 100). Larval shedding one week before necropsy ranged from 2 to 2770 larvae per gram faeces (LPG) (mean 1375 LPG). The study was conducted under the Danish experimental animal licence no. 2005/561-1060.

Time points were selected in analogy to Tritten et al. [32] in order to obtain serum profiles before infection (day 0; d0), during prepatency (day 35 dpi; d35), during patency (day 84 dpi; d84), and at a time point of chronic infection (day 154 dpi; d154, from 5 juvenile foxes only). For each fox, d0 represented the baseline time point. Sera samples had been stored at −20 °C for 13 years prior to the use for this study.

### 4.2. Serum Sample Preparation

From each fox serum sample, 200 μL was prepared as described by Tritten et al. [32]. Serum samples were processed using the ProteoMiner small capacity kit (Bio-Rad) for protein enrichment according to the manufacturer’s instructions. Proteins were then precipitated using a trichloroacetic acid (TCA) protocol. Briefly, protein eluates were diluted in dH_2_O, sodium deoxycholate 0.15%, and TCA 72%, incubated and centrifuged. Pellets were washed three times with ice-cold acetone and centrifuged. Samples were air-dried and resuspended in 50 µL 4% SDS, Tris-HCl 0.1 M, pH = 7.6, 0.1 M DTT and heated at 95 °C for 5 min. Protein content was measured by a Qubit protein assay (Thermo Fisher Scientific). Next, 30 µg of protein were further processed by filter-aided sample preparation [52]: samples were mixed with 8 M urea/100 mM Tris-HCl pH = 8.2, loaded onto Microcon 30 filter units (Millipore) and centrifuged. Samples were washed with urea buffer and treated with 0.05 M iodoacetamide (IAA), incubated and centrifuged. This was followed by three urea buffer washes and two 0.5 M NaCl washes. Then, 0.05 M triethylammoniumbicarbonate and sequencing grade modified trypsin (Promega, V5113) were added and samples were incubated overnight. Samples were centrifuged and trifluoroacetic acid (TFA) 5% was added to adjust pH. Samples were then desalted using C18 stage tips [53]: stage tips were washed and equilibrated with 100% methanol, 60% acetonitrile (ACN)/0.1% TFA and 3% ACN/0.1% TFA. Samples were treated with 3% ACN/0.1% TFA, loaded onto columns and centrifuged. They were then washed twice with 3% ACN/0.1% TFA and eluted with 60% ACN/0.1% TFA. Samples were dried to completeness using a speed-vac and resuspended in 3% ACN/0.1% formic acid (FA).

### 4.3. LC-MS Analysis

Fox serum peptide samples were diluted in 3% ACN, 0.1% FA to 1 µg/μL and retention time normalization peptides (iRT, Biognosys) added (1:20). Samples were analysed in random order by data independent analysis (DIA) on a hybrid quadrupole-Orbitrap mass spectrometer (Q Exactive HF, Thermo Fisher Scientific), which was operated in line with an Acquity UHPLC M-class system (Waters) with a nanoEase M/Z Symmetry C18 trap column (100 A, 5 μm, 180 μm × 20 mm, Waters) and a nanoEase M/Z HSS C18 T3 analytical column (100 A, 1.8 μm, 75 μm × 250 mm Column, Waters). Then, 1 μL per sample was eluted and run on a linear gradient from 5% to 32% 0.1% FA in ACN over 120 min at 300 nl/min. A nano electrospray ionization (ESI) source (Digital PicoView 565, O/N: DPV-550-565, New Objective, Woburn, MA) and a 10 μm fused-silica spray tip emitter (New Objective, PN) ionized the peptides. MS1 scans covered 350–1800 *m/z* and were recorded in centroid mode. They were recorded in positive polarity, with a resolution of 60,000 and automated gain control (AGC) with a target value of 3 × 10^6^. The maximum injection time (maxIT) was 200 ms. MS1 scans were followed by 35 DIA scans in positive polarity with 30,000 resolution. The AGC target was 1 × 10^6^ and the maxIT 55 ms. DIA scans covered a mass range of 400–1100 *m/z* in 20 *m/z* isolation windows and were recorded in centroid mode. Fixed first mass was set to 100 *m/z* and isolated precursors were fragmented with higher-energy collisional dissociation (HCD) at a normalized collision energy (NCE) of 28.

Spectronaut (v. 13; Biognosys) was used for label-free protein quantification. DirectDIA analysis based on the UniProt proteome of *Vulpes vulpes* (UP000286640, accessed: 29 June 2020), *Angiostrongylus costaricensis* (UP000050601, accessed: 9 January 2019), and *Angiostrongylus cantonensis* (UP000035642; accessed: 9 January 2019) was performed. *Angiostrongylus costaricensis* and *A. cantonensis* represented the two most closely related species to *A. vasorum* with available reference proteomes. These proteomes were included to identify circulating parasite proteins in serum samples. BGS factory settings were used for analysis. These settings included tryptic specificity, allowing two missed cleavages, carbamidomethyl as a fixed cysteine modification, and oxidation of methionine and protein N-terminal acetylation as variable modifications. Protein groups identified by a single peptide sequence were excluded. Single hit definition was defined by stripped sequence. FDR was set to 1% for peptide spectrum matches and proteins. The raw mass spectrometry data and Spectronaut outputs have been deposited to the ProteomeXchange Consortium via the PRIDE [54] partner repository (dataset identifier PXD028312).

### 4.4. Data Analysis

Data were exported from Spectronaut and analysed using the MSStats R package (v. 4.0.3) [55] for statistical relative quantification and significance analysis, as described by Tritten et al. [32]. Briefly, data were log2 transformed, normalized by equalizeMedians, and summarized by Tukey’s median polish. The model took repeated sampling from the same fox into account. Cut-offs for significant protein increase or decrease were defined by an adjusted *p*-value ≤ 0.05 and log2 FC ≥  1, or ≤ −1. For further analysis, specific human orthologues were retrieved via NCBI blastp for increased or decreased proteins, as defined by the cut-offs, since foxes are not well characterized. E values < 1 × 10^−20^ were considered high-quality matches. Orthologues were converted to gene symbols using DAVID (v. 6.8) [56] and analysed with Enrichr [57] for gene set enrichment analysis to obtain pathway and gene ontology terms.

### 4.5. Qualitative Comparison of Serum Alterations of Experimentally Infected Foxes and Dogs

Fox results were qualitatively compared to results obtained from dog samples from a previous study [32] in order to compare host-specific responses in these two animal species. Therefore, the same methods and similar time points were used to analyse serum samples of *A. vasorum* experimentally infected dogs and foxes. Briefly, serum samples (stored for 9 years) of 8 *A. vasorum* experimentally infected dogs from 3 time points were analysed: 7 days before infection (d-7), 34 days (d34) and 75 days (d75) after inoculation. From two dogs, two further time points were available and analysed (104 and 230 dpi; d104 and d230). Samples were enriched, precipitated, digested and analysed by LC-MS (DIA). Data were analysed in Proteome Discoverer and Spectronaut and further analysed using MSStats and STRING [32]. Protein abundance increase and decrease in fox time point comparisons were compared to the ones of dogs: d35 vs. d0 of foxes was compared to d34 vs. d-7 of dogs, foxes’ d84 vs. d0 was compared to d75 vs. d-7 in dogs, and d154 vs. d0 of foxes compared to both d104 and d230 vs. d-7 obtained from two dogs.

## 5. Conclusions

Serum proteins involved in the immune and inflammatory response are strongly increased upon experimental *A. vasorum* infection in farm foxes. Several altered proteins, including both increased and decreased levels of proteins interfering with coagulation, led to enriched coagulation pathways, suggesting simultaneous activation and suppression of blood clotting in foxes, either induced through the parasite itself or the host immune response. Considering the overall changes upon infection in foxes, the observed serum alterations are indicative of some disease development in foxes, although no clinical manifestations were observed in the animals of the present study. The qualitative comparison to dog serum data suggests some parallel serum alterations in foxes, encompassing abundance increase in proteins involved in inflammation or immune response and decrease in coagulation proteins. However, a strong increase in bone marrow proteoglycan was only observed in foxes, suggesting that foxes develop an increased innate immune response upon *A. vasorum* infection compared to dogs. This may imply that foxes are more tolerant to *A. vasorum* infection compared to dogs, reflecting a longer evolutionary host–parasite adaptation in foxes, and also supporting their important role as reservoir hosts under field conditions.

## Figures and Tables

**Table 1:** Top 10 gene ontology biological processes and Reactome pathways (obtained using human orthologues) for increased serum proteins of *Angiostrongylus vasorum* experimentally infected foxes in time point comparisons.

Rank	d35 vs. d0				d84 vs. d0				d154 vs. d0			
	Biological Process		Pathway		Biological Process		Pathway		Biological Process		Pathway	
	Term	Adjusted *p*-Value	Term	Adjusted *p*-Value	Term	Adjusted *p*-Value	Term	Adjusted *p*-Value	Term	Adjusted *p*-Value	Term	Adjusted *p*-Value
1	antibacterial humoral response (GO:0019731)	7.97 × 10^−4^	Extracellular matrix organization	0.0033	neutrophil mediated immunity (GO:0002446)	1.29 × 10^−10^	Extracellular matrix organization	1.54 × 10^−6^	neutrophil mediated immunity (GO:0002446)	2.32 × 10^−13^	Extracellular matrix organization	4.58 × 10^−13^
2	extracellular matrix organization (GO:0030198)	0.0016			neutrophil activation involved in immune response (GO:0002283)	1.70 × 10^−10^	Elastic fibre formation	1.28 × 10^−4^	neutrophil activation involved in immune response (GO:0002283)	2.93 × 10^−13^	Elastic fibre formation	1.86 × 10^−5^
3	neutrophil mediated immunity (GO:0002446)	0.0038			neutrophil degranulation (GO:0043312)	2.96 × 10^−10^	Molecules associated with elastic fibres	0.0011	neutrophil degranulation (GO:0043312)	4.92 × 10^−13^	Molecules associated with elastic fibres	9.80 × 10^−5^
4	neutrophil activation involved in immune response (GO:0002283)	0.0045			extracellular matrix organization (GO:0030198)	4.42 × 10^−5^	Keratan sulphate degradation	0.0023	extracellular matrix organization (GO:0030198)	9.29 × 10^−11^	Metabolism of carbohydrates	1.39 × 10^−4^
5	neutrophil degranulation (GO:0043312)	0.0056			sulphur compound catabolic process (GO:0044273)	0.0035	Platelet degranulation	0.0057	antibacterial humoral response (GO:0019731)	9.13 × 10^−6^	Response to elevated platelet cytosolic Ca^2+^	1.41 × 10^−4^
6	cell-matrix adhesion (GO:0007160)	0.0061			regulated exocytosis (GO:0045055)	0.0041	Response to elevated platelet cytosolic Ca^2+^	0.0059	cell-matrix adhesion (GO:0007160)	1.18 × 10^−4^	Platelet degranulation	1.54 × 10^−4^
7	regulation of endothelial cell apoptotic process (GO:2000351)	0.0088			antibacterial humoral response (GO:0019731)	0.0042	Common Pathway of Fibrin Clot Formation	0.0092	defence response to bacterium (GO:0042742)	0.0081	Keratan sulphate degradation	0.0034
8	cellular macromolecular complex assembly (GO:0034622)	0.0091			keratan sulphate catabolic process (GO:0042340)	0.0045	Metabolism of proteins	0.018	platelet degranulation (GO:0002576)	0.0092	Axon guidance	0.0035
9	regulation of extrinsic apoptotic signalling pathway via death domain receptors (GO:1902041)	0.010			cellular protein metabolic process (GO:0044267)	0.0097	Keratan sulphate on keratin metabolism	0.022	keratan sulphate catabolic process (GO:0042340)	0.010	Non-integrin membrane-ECM interactions	0.0059
10	retina homeostasis (GO:0001895)	0.010			glycosaminoglycan catabolic process (GO:0006027)	0.014	Formation of Fibrin Clot (Clotting Cascade)	0.037	sulphur compound catabolic process (GO:0044273)	0.011	Degradation of the extracellular matrix	0.016

**Table 2:** Top 10 gene ontology biological processes and Reactome pathways (obtained using human orthologues) for decreased serum proteins of *Angiostrongylus vasorum* experimentally infected foxes in time point comparisons.

Rank	d35 vs. d0				d84 vs. d0				d154 vs. d0			
	Biological Process		Pathway		Biological Process		Pathway		Biological Process		Pathway	
	Term	Adjusted *p*-Value	Term	Adjusted *p*-Value	Term	Adjusted *p*-Value	Term	Adjusted *p*-Value	Term	Adjusted *p*-Value	Term	Adjusted *p*-Value
1	positive regulation of establishment of protein localization to telomere (GO:1904851)	7.26 × 10^−5^	Prefoldin mediated transfer of substrate to CCT/TriC	3.12 × 10^−11^	positive regulation of establishment of protein localization to telomere (GO:1904851)	4.79 × 10^−4^	Prefoldin mediated transfer of substrate to CCT/TriC	6.89 × 10^−12^	regulation of protein localization to Cajal body (GO:1904869)	3.05 × 10^−5^	Prefoldin mediated transfer of substrate to CCT/TriC	4.54 × 10^−9^
2	regulation of protein localization to Cajal body (GO:1904869)	7.27 × 10^−5^	Formation of tubulin folding intermediates by CCT/TriC	3.74 × 10^−11^	regulation of protein localization to Cajal body (GO:1904869)	4.79 × 10^−4^	Formation of tubulin folding intermediates by CCT/TriC	7.48 × 10^−12^	positive regulation of protein localization to Cajal body (GO:1904871)	6.10 × 10^−5^	Formation of tubulin folding intermediates by CCT/TriC	4.95 × 10^−9^
3	regulation of establishment of protein localization to telomere (GO:0070203)	7.78 × 10^−5^	Cooperation of Prefoldin and TriC/CCT in actin and tubulin folding	6.36 × 10^−11^	regulation of establishment of protein localization to telomere (GO:0070203)	5.12 × 10^−4^	Cooperation of Prefoldin and TriC/CCT in actin and tubulin folding	1.73 × 10^−11^	positive regulation of telomerase RNA localization to Cajal body (GO:1904874)	3.89 × 10^−4^	Haemostasis	6.69 × 10^−9^
4	positive regulation of protein localization to chromosome, telomeric region (GO:1904816)	8.55 × 10^−5^	Chaperonin-mediated protein folding	4.44 × 10^−8^	positive regulation of protein localization to chromosome, telomeric region (GO:1904816)	5.63 × 10^−4^	Chaperonin-mediated protein folding	3.99 × 10^−8^	regulation of telomerase RNA localization to Cajal body (GO:1904872)	4.32 × 10^−4^	Cooperation of Prefoldin and TriC/CCT in actin and tubulin folding	8.48 × 10^−9^
5	positive regulation of establishment of protein localization (GO:1904951)	9.50 × 10^−5^	Protein folding	5.17 × 10^−8^	positive regulation of establishment of protein localization (GO:1904951)	6.25 × 10^−4^	Protein folding	4.94 × 10^−8^	positive regulation of protein localization to nucleus (GO:1900182)	4.72 × 10^−4^	Folding of actin by CCT/TriC	1.09 × 10^−5^
6	positive regulation of protein localization to Cajal body (GO:1904871)	1.45 × 10^−4^	Folding of actin by CCT/TriC	1.55 × 10^−5^	positive regulation of protein localization to Cajal body (GO:1904871)	9.58 × 10^−4^	Post-chaperonin tubulin folding pathway	8.64 × 10^−6^	platelet degranulation (GO:0002576)	4.83 × 10^−4^	Chaperonin-mediated protein folding	1.54 × 10^−5^
7	positive regulation of telomerase RNA localization to Cajal body (GO:1904874)	1.68 × 10^−4^	Cell-extracellular matrix interactions	6.20 × 10^−5^	positive regulation of telomerase RNA localization to Cajal body (GO:1904874)	0.0011	Folding of actin by CCT/TriC	8.78 × 10^−5^	regulation of lipoprotein lipase activity (GO:0051004)	5.10 × 10^−4^	Protein folding	2.02 × 10^−5^
8	regulation of telomerase RNA localization to Cajal body (GO:1904872)	2.63 × 10^−4^	Post-chaperonin tubulin folding pathway	1.49 × 10^−4^	regulation of telomerase RNA localization to Cajal body (GO:1904872)	0.0017	Cell-extracellular matrix interactions	3.56 × 10^−4^	hydrogen peroxide metabolic process (GO:0042743)	5.83 × 10^−4^	Platelet degranulation	2.32 × 10^−5^
9	positive regulation of telomere maintenance via telomerase (GO:0032212)	0.0014	Association of TriC/CCT with target proteins during biosynthesis	7.78 × 10^−4^	regulation of lipoprotein lipase activity (GO:0051004)	0.0021	Platelet degranulation	0.0034	regulated exocytosis (GO:0045055)	7.10 × 10^−4^	Response to elevated platelet cytosolic Ca^2+^	2.84 × 10^−5^
10	positive regulation of telomere maintenance via telomere lengthening (GO:1904358)	0.0015	Cooperation of PDCL (PhLP1) and TRiC/CCT in G-protein beta folding	8.78 × 10^−4^	acylglycerol homeostasis (GO:0055090)	0.0026	Response to elevated platelet cytosolic Ca^2+^	0.0037	chylomicron remodelling (GO:0034371)	0.0019	Cell-extracellular matrix interactions	4.23 × 10^−5^

## Data Availability

The raw mass spectrometry data and Spectronaut outputs have been deposited to the ProteomeXchange Consortium via the PRIDE [54] partner repository (dataset identifier PXD028312). The datasets supporting the results and conclusions of this article are included within the article and its supplementary files. Dog serum data used for comparison of fox serum alteration to data from experimentally infected dogs are available from Tritten et al. [32] at https://doi.org/10.1038/s41598-020-79459-9 (including supplementary information). The R code (MSstats) and further data are available from the corresponding authors upon request.

## Supplementary Materials

The following are available online at https://www.mdpi.com/article/10.3390/pathogens10111513/s1. Supplementary Figure S1 displays a heat map of identified proteins from all examined fox serum samples. Supplementary Table S1 contains a detailed list with all significant increased or decreased fox serum proteins of all time point comparisons. Supplementary Table S2 contains all significant biological processes and enriched pathways obtained from either increased or decreased proteins.
