# Supplementary figures and images for: Fox Serum Proteomics Analysis Suggests Host-Specific Responses to Angiostrongylus vasorum Infection in Canids
Source: Pathogens. 2021 Nov 19;10(11):1513. doi: 10.3390/pathogens10111513 (PMC8623225; doi:10.3390/pathogens10111513)

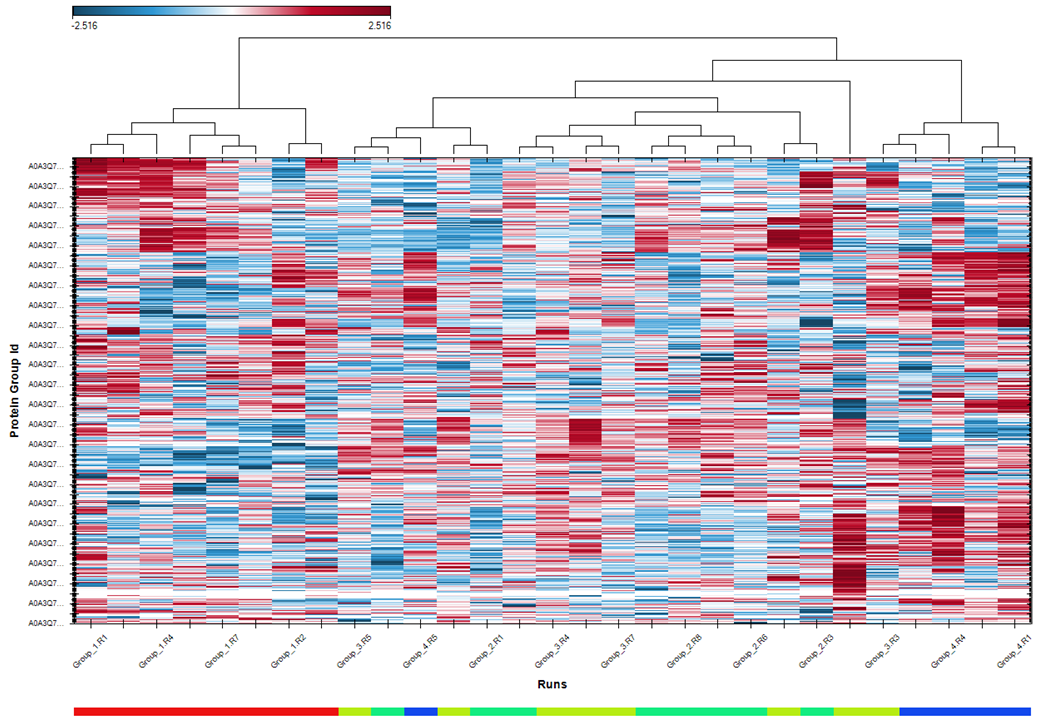

Supplement: Supplementary file 1 [file pathogens-10-01513-s001.zip › Supplementary files/Supplementary Figure S1.tif]
